# Leg ulcer with long‐term hydroxyurea use

**DOI:** 10.1002/ccr3.3991

**Published:** 2021-02-24

**Authors:** Mohammad Ammad Ud Din, Syed Ather Hussain, Saad Jamshed

**Affiliations:** ^1^ Department of Internal Medicine Rochester General Hospital Rochester NY USA; ^2^ Department of Hematology & Oncology Rochester General Hospital Rochester NY USA

**Keywords:** essential thrombocytosis, hematology, hydroxyurea

## Abstract

Long‐term use of hydroxyurea can cause leg ulcers which usually do not heal unless the drug is discontinued. Patients should be counseled regarding alternative lines of treatment like anagrelide and pegylated‐interferon.

## Graphical abstract

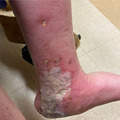

A 63‐year‐old nondiabetic male with a history of essential thrombocytosis presented with a painful nonhealing ulcer on the medial aspect of his lower left leg for the past 3 weeks (Figure 1). He denied any fevers, chills, or trauma to the leg. Laboratory evaluation showed normal hemoglobin and white blood cell count, but his platelet count was elevated at 1300 × 103/uL. A computed tomography (CT) angiogram of the lower extremities revealed no anomalies. His leg ulcer was thought to be secondary hydroxyurea use as he was taking 1000 mg twice daily of hydroxyurea for the past 4 years for essential thrombocytosis. His symptoms improved with the drug discontinuation. Anagrelide and pegylated‐interferon were recommended as alternative treatment options, but he chose to pursue homeopathic care.

**FIGURE 1:**
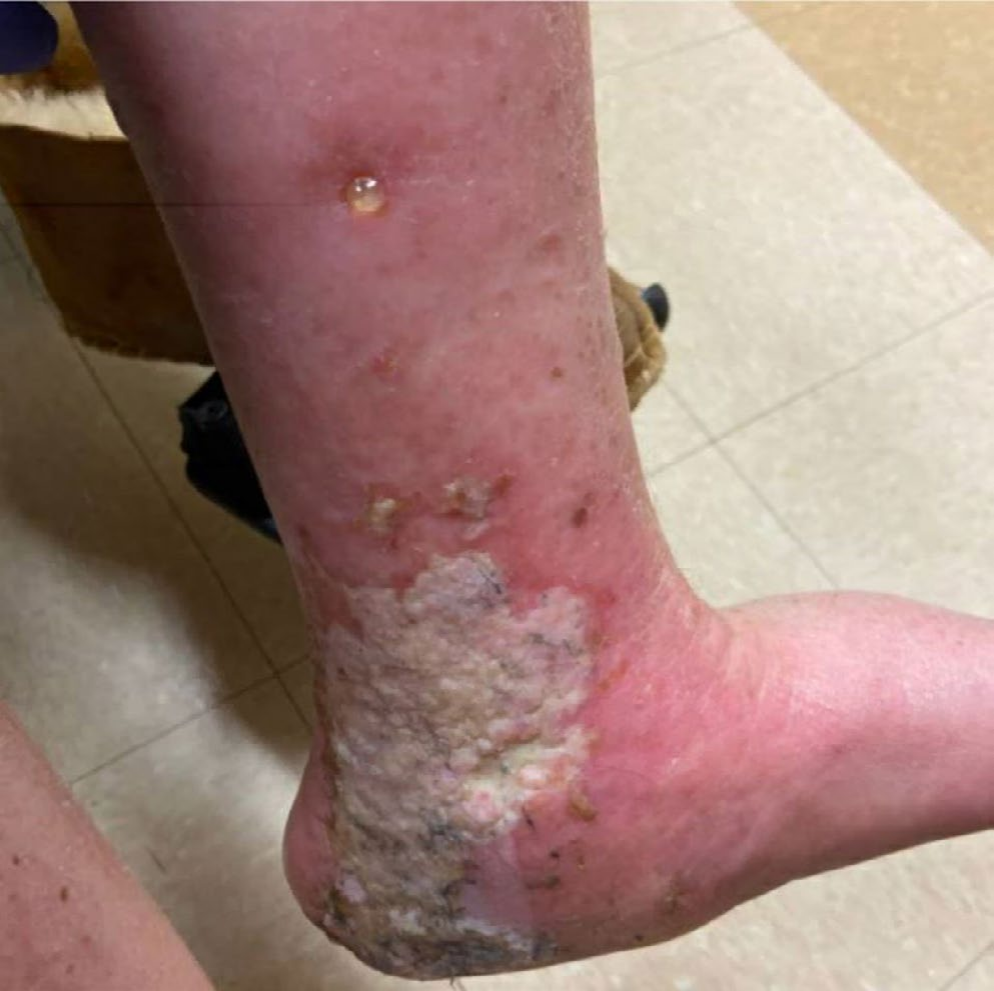
A large nonhealing ulcer on the left malleolus. Characteristic atrophie blanche (white stellate scarring) and surrounding erythema can be seen

Leg ulcers can result from long‐standing use of hydroxyurea in about 10% of the patients.^1^ These ulcers are painful and often emerge spontaneously in the malleolar region. They occur as a result of the cytotoxic action of the drug causing inhibition of the DNA synthesis in the basal keratinocytes leading to disruption in the production of skin collagen. The ulcers do not respond to traditional wound care, and healing requires complete cessation of hydroxyurea.^2^

## CONFLICT OF INTEREST

The authors declared no potential conflicts of interest with respect to the research, authorship, and/or publication of this manuscript.

## AUTHOR CONTRIBUTIONS

MAUD and SAH: performed literature search and wrote the manuscript. SJ: served as the primary hematologist on the case and critically reviewed the manuscript and made final edits prior to the submission.

## ETHICAL APPROVAL

This report for a clinical image was conducted in accordance with the Declaration of Helsinki. The collection and evaluation of all protected patient health information was performed in a Health Insurance Portability and Accountability (HIPAA) complaint manner. A formal informed consent was obtained from the patient prior to the publication of this article.

## Data Availability

Data sharing not applicable to this article as no data sets were generated or analyzed during the current study.
